# Editorial: Advancing motor coordination therapy for children with developmental disorders

**DOI:** 10.3389/fped.2026.1922029

**Published:** 2026-07-21

**Authors:** Antonella Muscella

**Affiliations:** Department of Biological and Environmental Science and Technologies (DiSTeBA), University of Salento, Lecce, Italy

**Keywords:** developmental disorders, ecological dynamics, motor competence, motor coordination, physical fitness

Motor coordination in childhood is a systems-level emergent property arising from the dynamic interaction between cortical network organization, sensorimotor integration loops, peripheral physiological states, and environmental affordances (1). In neurodevelopmental conditions—such as autism spectrum disorder (ASD), developmental coordination disorder (DCD), attention-deficit/hyperactivity disorder (ADHD), and cerebral palsy (CP)—motor impairment is not merely a downstream consequence of cognitive dysfunction, but a core organizational phenotype reflecting atypical development of distributed brain–body networks (Cao et al.; Shen et al.). Across the contributions to this Research Topic, a convergent conceptual shift emerges motor coordination is the behavioral expression of hierarchical neurobiological systems integrating prediction, action selection, metabolic readiness, and social coupling.

Emerging neuroimaging evidence frames pediatric motor impairment as a manifestation of disrupted large-scale brain network organization rather than localized pathway dysfunction (2). In ASD, altered functional connectivity across sensorimotor and association networks suggests that motor difficulties arise from impaired integration within distributed systems responsible for sensorimotor prediction, state estimation, and action selection (Cao et al.). Within predictive coding architectures, motor behavior reflects the precision-weighting of internal models; reduced fidelity in representing bodily and environmental states leads to increased variability, temporal inefficiency, and diminished adaptive flexibility (3, 4).

This neural-level interpretation aligns with evidence highlighting intermediate physiological mechanisms linking motor capacity to functional behavior. Rather than exerting a direct influence on social functioning, motor coordination operates through a mediating pathway involving health-related physical fitness, suggesting a latent physiological interface through which neuromotor capacity is translated into socially relevant outcomes (Shen et al.). This intermediate layer reflects the coupling between energetic constraints, neuromuscular efficiency, and action–perception integration processes, which jointly determine the effective expression of motor competence in ecological contexts (5).

Chen et al. (6) extend this perspective by demonstrating that pediatric motor development is not adequately described by linear constructs. Using data-driven clustering, they identify discrete latent physical fitness phenotypes in preschool children, indicating that motor competence is organized into structured states rather than continuous gradients. This aligns with developmental systems and ecological dynamics frameworks, where behavior emerges from constrained state spaces shaped by interacting organismic and environmental factors (7, 8). Within this framework, anthropometric determinants such as central adiposity and pelvic morphology act as structural constraints defining these emergent phenotypic boundaries (6).

Importantly, this interpretation is reinforced by a second modeling layer linking morphology, motor coordination, and fitness. Zhao et al. (9) demonstrate that body mass index (BMI) and motor coordination are jointly structured through health-related physical fitness, which acts as a central mediating variable in preschool children. This supports the existence of a hierarchical physiological interface through which structural and motor variables converge to shape developmental trajectories.

At the molecular level, Zhong et al. provide evidence that circulating microRNAs exhibit high diagnostic accuracy in ADHD, reinforcing the concept that neurodevelopmental phenotypes can be partially captured through peripheral epigenetic signatures reflecting central dysregulation. This adds a molecular dimension to the multi-layered architecture of motor and cognitive development.

Complementing neural and molecular perspectives, systemic biological regulation plays a critical role in shaping motor developmental outcomes. Endocrine modulation via growth hormone (GH) pathways influences neuromuscular maturation and functional readiness beyond its canonical effects on linear somatic growth. As synthesized by Shen et al., long-acting GH (LAGH) formulations—particularly PEGylated GH (PEG-LAGH)—are associated with superior first-year growth outcomes compared to conventional daily injections, including higher height velocity and improved height standard deviation scores. These effects are attributed to optimized pharmacokinetic profiles characterized by more physiological pulse-amplitude exposure patterns, which may enhance JAK2–STAT5 signaling dynamics and, indirectly, influence systemic conditions relevant to neuromuscular development and functional capacity.

In parallel, immune–neural coupling represents a further layer of systemic modulation relevant to pediatric motor outcomes. Zhao et al. demonstrate that improved health status associated with routine immunization is linked to better longitudinal functional trajectories in children with cerebral palsy. Over a 24-month follow-up, vaccinated children showed higher probabilities of clinical improvement, with 33.7% achieving at least a one-level gain in Gross Motor Function Classification System (GMFCS) and 38.1% in Communication Function Classification System (CFCS), compared with lower proportions in unvaccinated peers. Notably, these effects appear more pronounced for global motor and communicative domains than for fine motor or swallowing-related functions, suggesting a preferential impact on distributed neural systems underlying higher-order functional integration rather than localized motor subsystems.

León-Bravo et al. complement this multi-system architecture by demonstrating that short-term somatosensory interventions, such as craniosacral therapy (CST), can transiently modulate developmental scores (driving an 8.4-point increase in adjusted BDI-2 composite scores) and reduce persistent primitive reflex expression (such as the ATNR and Moro reflex). This highlights residual functional plasticity within low-level sensorimotor regulation systems under standardized clinical conditions.

Within ecological frameworks, motor competence is shaped by context-dependent constraints. The cross-cultural validation of the DCDDaily-Q underscores that functional motor behavior emerges from organism–environment coupling shaped by cultural practices and task demands (Huang et al.). Interventions, therefore, must operate as controlled perturbations of this complex adaptive system.

Immersive virtual reality (VR)-based serious games enhance motor engagement and independent communication drives by embedding action within enriched predictive simulations (Ma and Song). Similarly, multi-sport training, such as the 12-week Ball Combination Training Program (BCTP) designed by Mao et al., increases behavioral dimensionality and system adaptability, successfully halting unhealthy BMI progression while simultaneously upgrading speed, agility, and upper-limb explosive power.

Furthermore, these systems exhibit remarkable stability across external environmental fluctuations. Music–motor interventions demonstrate robust behavioral adjustments independent of time-of-day constraints, suggesting the modulation of stable neural attractor states rather than circadian-dependent mechanisms (Kanzari et al.).

However, unlocking these adaptations requires precise quantitative dosing. Nonlinear meta-regression modeling by Gao et al. reveals that exercise interventions for balance performance follow strict optimization trajectories, pinpointing the maximal therapeutic benefit at an optimal physical training volume of exactly 717 MET·min/week. Superior sensorimotor returns are unlocked when training adheres to structural parameters of at least 3 sessions per week and a session duration exceeding 60 min, beyond which adaptive returns diminish.

Finally, motor imagery represents a cognitive-level mechanism supporting internal simulation and refinement of predictive motor models, extending rehabilitation targets beyond overt movement execution (Chi et al.). At a broader epidemiological level, bibliometric mapping of adolescent idiopathic scoliosis (AIS) treatment from 2009 to 2024 highlights a clear clinical shift toward precision-oriented, data-driven stratification integrating Cobb angle measurements, non-surgical orthotic compliance modeling, and long-term patient-reported prognoses (Zheng et al.), mirroring the precision trends observed in pediatric motor neuroscience.

Collectively, the studies included in this Research Topic converge toward a unified reconceptualization of pediatric motor coordination as an emergent property of interacting neural, physiological, and environmental systems. Across molecular, neurophysiological, behavioral, and ecological levels, motor development is understood as the product of hierarchical interactions linking predictive brain dynamics, systemic biological regulation, and context-dependent constraints.

**Figure 1:**
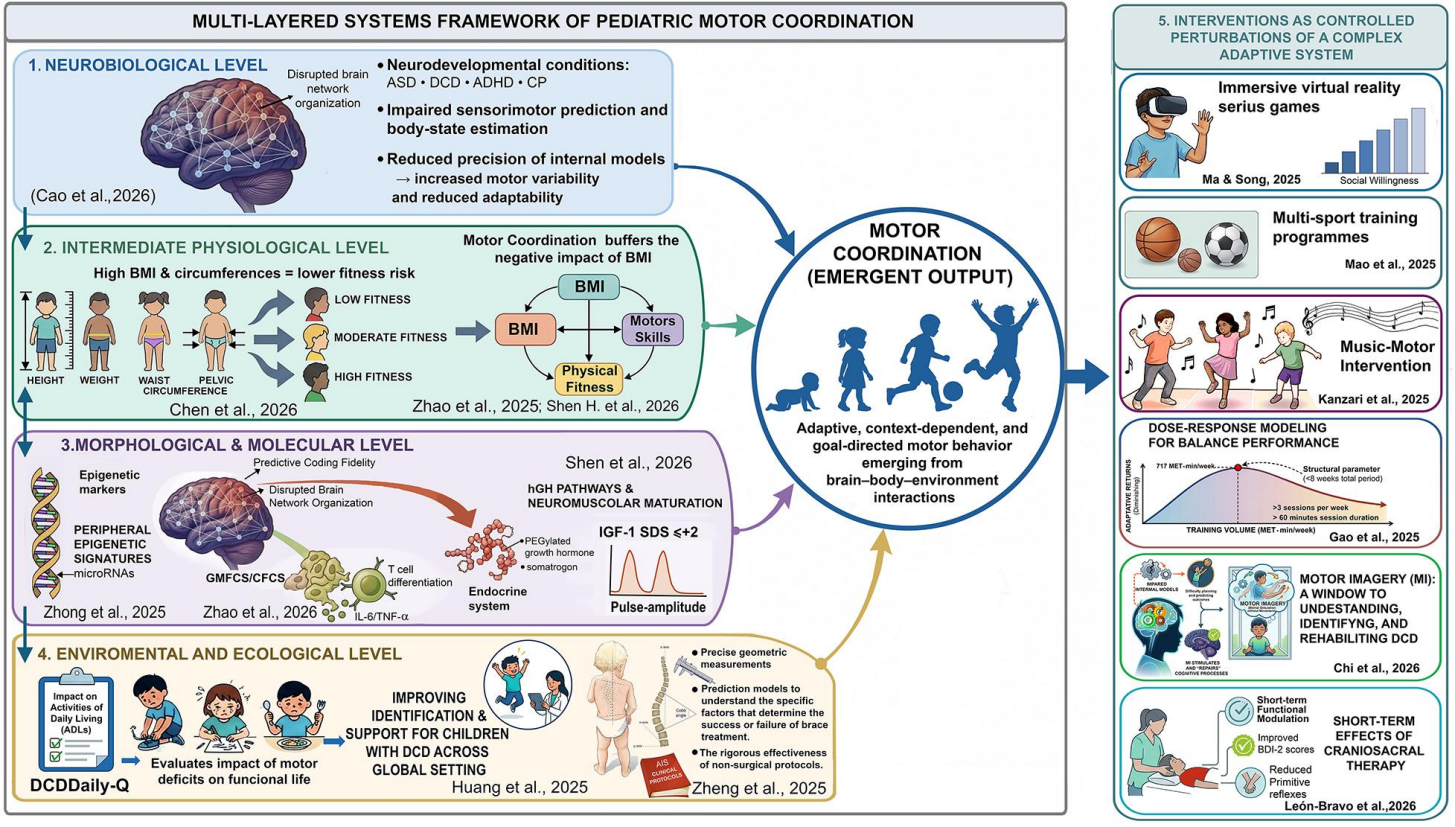
Multi-layered systems framework of pediatric motor coordination. Motor coordination is conceptualized as an emergent outcome arising from dynamic interactions among neurobiological, physiological, morphological and molecular, environmental, and ecological factors. The framework illustrates how individual characteristics, biological mechanisms, contextual influences, and targeted interventions collectively shape motor development and performance in children. Intervention approaches—including virtual reality, serious games, multi-sport programs, music-motor training, motor imagery, and other therapeutic strategies—are presented as controlled perturbations capable of promoting motor adaptation and skill acquisition. References within the figure represent illustrative studies supporting each framework component.

This integrative perspective challenges reductionist models of pediatric motor impairment and supports a shift toward multidimensional frameworks where motor behavior reflects the observable output of distributed brain–body–environment couplings (Figure 1). Effective intervention strategies must simultaneously engage neural predictive systems, physical fitness mediators, and enriched ecological learning environments. Future research should prioritize longitudinal, multi-scale, and computationally informed approaches capable of capturing developmental transitions across neural, physiological, and behavioral domains to formalize how motor competence emerges from dynamically constrained neurodevelopmental systems.
